# Post-Stroke Epilepsy After Pediatric Arterial Ischemic Stroke: Incidence and Risk Factors—A Narrative Review

**DOI:** 10.3390/brainsci16090907

**Published:** 2026-08-25

**Authors:** Federica Teutonico, Irene Puma, Aglaia Vignoli

**Affiliations:** 1 Childhood and Adolescence Neurology and Psychiatry Unit, ASST GOM Niguarda, 20162 Milano, Italy; federica.teutonico@ospedaleniguarda.it; 2 Department of Health Sciences, University of Milano, 20122 Milano, Italy; irene.puma@unimi.it

**Keywords:** pediatric stroke, post-stroke epilepsy, acute symptomatic seizures, arterial ischemic stroke, epilepsy risk factors

## Abstract

**Highlights:**

**What are the main findings?**

- Approximately one in four children who experience an arterial ischemic stroke will eventually develop epilepsy, although reported incidence varies widely across studies (7% to >40%).
- Acute symptomatic seizures, particularly very early seizures and status epilepticus, are the strongest predictors of post-stroke epilepsy (≈4-fold increased risk), while age, cortical involvement, greater lesion burden, persistent neurological deficits, and possibly genetic and biological factors further increase risk.

**What are the implications of the main findings?**

- Children presenting with acute symptomatic seizures and other high-risk clinical or radiological features should undergo closer long-term surveillance for the early identification and management of post-stroke epilepsy.
- Improved risk stratification may support personalized follow-up strategies and stimulate research into preventive interventions targeting potentially modifiable factors, such as vitamin D deficiency, and genetic susceptibility.

**Abstract:**

**Background/Objectives**: Post-stroke epilepsy represents one of the most frequent and impactful long-term sequelae of pediatric arterial ischemic stroke. Children are particularly vulnerable to developing seizures and epilepsy after stroke, likely because the immature brain responds differently to ischemic injury than the adult brain. The purpose of this narrative review is to summarize the current evidence regarding the epidemiology, risk factors, mechanisms, and clinical implications of post-stroke epilepsy following pediatric arterial ischemic stroke. **Methods**: We conducted a narrative review to examine the incidence, determinants, risk stratification, and long-term outcomes of post-stroke epilepsy following childhood arterial ischemic stroke beyond the neonatal period. **Results**: The reported incidence of post-stroke epilepsy in children varies considerably across studies, ranging from approximately 7% to over 40%. However, recent data suggest that roughly one in four children who experience an arterial ischemic stroke will eventually develop epilepsy, highlighting the substantial long-term burden of this condition. Acute symptomatic seizures, particularly very early seizures and acute status epilepticus, consistently emerge as the strongest predictors of subsequent epilepsy, with recent pediatric meta-analytic data suggesting an approximately fourfold increased risk of post-stroke epilepsy. Cortical involvement and persistent neurological deficits also represent robust predictors of post-stroke epilepsy (PSE), whereas younger age at stroke onset, multifocal infarctions, greater infarct burden, anterior circulation involvement, and focal cerebral arteriopathy have been associated with an increased risk. Emerging evidence further suggests a possible contribution of genetic susceptibility and potentially modifiable biological factors, such as vitamin D deficiency, to epileptogenic risk. **Conclusions**: This narrative review summarizes current evidence regarding the incidence, determinants, and pathophysiological mechanisms of post-stroke epilepsy following childhood arterial ischemic stroke, highlighting established and emerging risk factors and their implications for long-term risk stratification, clinical management, and future preventive strategies.

## 1. Introduction

Pediatric arterial ischemic stroke (AIS) is increasingly recognized as an important cause of long-term neurological morbidity and disability. Although less frequent than adult stroke, AIS in children is associated with substantial mortality and significant long-term neurological sequelae, including motor deficits, cognitive impairment, behavioral disturbances, language dysfunction, and epilepsy [1,2,3]. Current epidemiological studies estimate an incidence ranging from 1 to 13 per 100,000 children per year, making pediatric stroke one of the leading causes of acquired neurological disability during childhood. Among the long-term consequences of AIS, epilepsy is one of the most common and clinically significant complications. The occurrence of seizures can have a substantial impact on neurodevelopment, academic achievement, psychosocial well-being, quality of life, and long-term cognitive outcomes. Compared with adults, children appear to be particularly susceptible to post-stroke epileptogenesis, likely reflecting the unique characteristics of the developing brain, including heightened excitatory neurotransmission, immature inhibitory networks, and increased neuronal plasticity [4,5,6].

Both experimental and clinical studies suggest that ischemic injury in the developing brain initiates a series of interconnected processes, including excitotoxicity, neuroinflammation, gliosis, synaptic remodeling, and large-scale network reorganization. These changes may continue well beyond the acute phase of stroke and are thought to play a key role in epileptogenesis. Over time, they can promote the formation of hyperexcitable neuronal networks that ultimately give rise to recurrent unprovoked seizures [7]. Despite increasing recognition of post-stroke epilepsy (PSE) as a major long-term sequela of pediatric AIS, estimates of incidence and risk factors remain heterogeneous across studies.

Differences in stroke subtype, age groups, definitions of seizures, duration of follow-up, and neuroimaging characteristics contribute to variability in the literature. Recent systematic reviews and population-based cohorts have nevertheless improved understanding of the determinants of epileptogenesis after pediatric AIS [1,2,6,8].

This narrative review summarizes current evidence regarding the incidence, determinants, and pathophysiological mechanisms of PSE after pediatric AIS beyond the neonatal period, highlighting implications for long-term clinical management and future strategies.

## 2. Materials and Methods

A focused narrative review was conducted to summarize the available evidence on the incidence, risk factors, pathophysiological mechanisms, and long-term outcomes of PSE following childhood arterial ischemic stroke. A narrative approach was selected because of the considerable heterogeneity of the available literature in terms of study design, patient populations, seizure and epilepsy definitions, follow-up duration, and outcome assessment.

A literature search was performed in PubMed/MEDLINE up to 20 February 2026. Eligible publications included peer-reviewed articles published in English from 1 January 2004 to 20 February 2026. The search strategy combined relevant keywords and Medical Subject Headings (MeSH), including “pediatric stroke,” “childhood arterial ischemic stroke,” “post-stroke epilepsy,” “acute symptomatic seizures,” “seizures after stroke,” “epileptogenesis,” and “risk factors,” using the Boolean operators AND and OR as appropriate.

Titles and abstracts were screened for relevance by the authors. Full texts of potentially eligible articles were subsequently reviewed, and additional publications were identified through manual screening of the reference lists of the selected studies and relevant reviews. Priority was given to original pediatric cohort studies, population-based investigations, and experimental studies addressing the incidence, clinical and neuroimaging predictors, pathophysiological mechanisms, or long-term outcomes of post-stroke epilepsy. Systematic reviews and consensus statements were used primarily to contextualize the available evidence and support definitions or general recommendations.

The review focused on arterial ischemic stroke occurring beyond the neonatal period. Studies exclusively including neonates aged 29 days or younger were excluded because neonatal stroke differs from childhood stroke in terms of developmental neurobiology, underlying etiologies, seizure susceptibility, and mechanisms of epileptogenesis. Studies including mixed neonatal and childhood populations were considered only when findings relevant to post-neonatal children could be separately identified or when they provided important contextual information; these limitations were explicitly acknowledged in the interpretation of the results.

Because this study was designed as a narrative rather than a systematic review, no formal quantitative synthesis, meta-analysis, or standardized risk-of-bias assessment was performed. Nevertheless, study design, sample size, population characteristics, duration of follow-up, and consistency of findings across studies were considered when evaluating the strength of the evidence.

## 3. Results

### 3.1. Definitions and Classification of Seizures After Pediatric Arterial Ischemic Stroke

**Figure 1:**
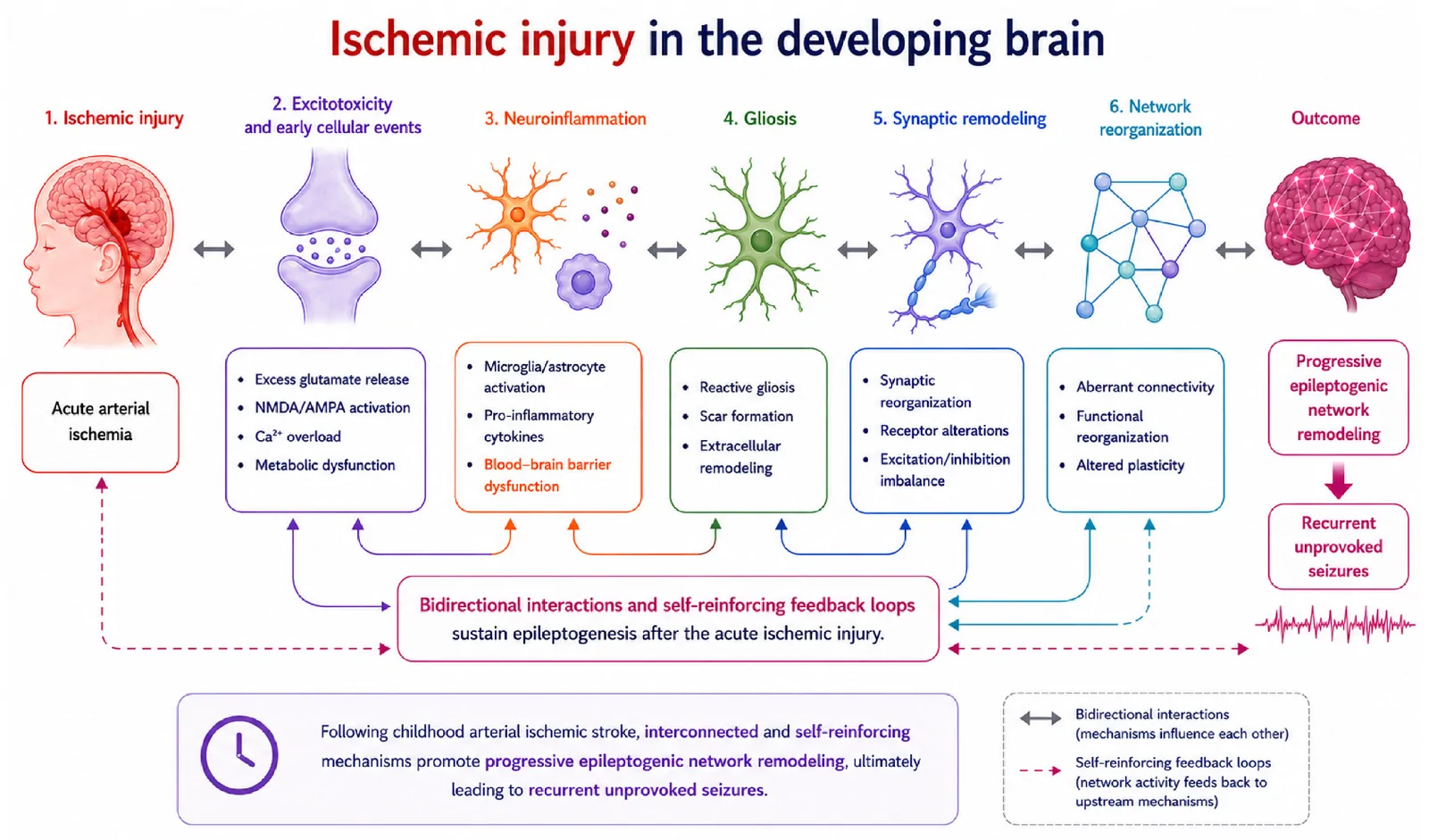
Proposed mechanisms underlying post-stroke epileptogenesis after childhood arterial ischemic stroke. Cerebral ischemia initiates a complex cascade involving excitotoxicity, neuroinflammation, gliosis, synaptic remodeling, and large-scale network reorganization. These mechanisms interact through multiple bidirectional feedback loops that may persist beyond the acute phase and progressively promote the formation of hyperexcitable epileptogenic networks capable of generating recurrent unprovoked seizures. Developmental factors may further modulate these processes in the immature brain.

Seizures occurring after pediatric stroke are commonly classified according to their timing in relation to the cerebrovascular event. Acute symptomatic seizures are defined as seizures occurring within 7 days of stroke onset [5]. Some studies further subdivide these events into very early seizures, which occur within the first 6 h, and early seizures, typically occurring within the first 24–48 h after stroke [9,10]. These seizures are believed to reflect acute metabolic and electrophysiological consequences of ischemic injury, including cytotoxic edema, glutamate-mediated excitotoxicity, ionic imbalance, and acute disruption of cortical networks. Late seizures, also known as remote symptomatic seizures, occur beyond the acute phase and are considered unprovoked. According to the International League Against Epilepsy (ILAE), PSE is diagnosed when recurrent unprovoked seizures develop following stroke, indicating the presence of a persistent epileptogenic condition [11]. The distinction between acute symptomatic seizures and remote unprovoked seizures is important from both a clinical and pathophysiological perspective. Acute symptomatic seizures are generally considered a direct consequence of the acute ischemic insult, arising from mechanisms such as excitotoxicity, ionic and metabolic disturbances, cytotoxic edema, altered neurotransmitter signaling, and transient disruption of neuronal membrane function. In contrast, remote unprovoked seizures and PSE are thought to result from a complex and dynamic process of epileptogenesis that evolves over time. Rather than representing a simple linear cascade, ischemia-induced excitotoxicity, neuroinflammation, gliosis, synaptic remodeling, and network reorganization interact through multiple bidirectional and self-reinforcing mechanisms that progressively promote the formation of hyperexcitable neuronal circuits capable of generating recurrent unprovoked seizures [12]. In the developing brain, enhanced neuronal plasticity and the ongoing maturation of inhibitory circuits may further modulate these processes (Figure 1).

### 3.2. Incidence of Seizures and Post-Stroke Epilepsy in Childhood

#### 3.2.1. Acute Symptomatic Seizures

Acute symptomatic seizures are common in pediatric AIS, with reported frequencies ranging from approximately 20% to over 60% across hospital-based and population-based cohorts [4,5,6,9,13]. In infants, seizures frequently represent the initial manifestation of stroke because focal neurological deficits may be subtle or difficult to recognize [3,4,5]. Several studies demonstrate that younger age at stroke onset and cortical involvement are strongly associated with acute seizure occurrence [4,6,9].

#### 3.2.2. Post-Stroke Epilepsy (PSE)

The reported cumulative incidence of PSE in childhood varies widely across studies, ranging from approximately 7% to 41% [1,2,6,14]. Despite the substantial heterogeneity across studies, the recent systematic review by Alqahtani and Makke estimated a pooled PSE incidence of 27.6% (95% CI 19.8–37.2%), although differences in study populations, follow-up duration, and outcome definitions should be considered when interpreting this estimate [8].

Large population-based studies have shown that approximately 10–15% of children develop epilepsy within the first 3–5 years following stroke [1,2]. The wide variability in reported incidence likely reflects differences in study populations, duration of follow-up, outcome definitions, and study design. Accordingly, incidence estimates should be interpreted in the context of the specific follow-up period and population assessed.

However, the risk is not limited to the early post-stroke period. Registry-based data indicate that the likelihood of developing epilepsy remains significantly increased for many years after the initial event. Although the highest risk is observed during the first months after stroke, an excess risk persists for decades, highlighting the long-term nature of post-stroke epileptogenesis in childhood [1,6]. Nationwide cohorts report cumulative incidences exceeding 25% after 20–30 years of follow-up, underscoring the long-term nature of post-stroke epileptogenesis [1,6].

### 3.3. Risk Factors for Post-Stroke Epilepsy

#### 3.3.1. Acute Symptomatic Seizures and Seizure Timing

Acute symptomatic seizures represent the strongest predictor of PSE in children [6,9,10,15,16,17]. Children who experience seizures during the acute phase of stroke have a substantially increased risk of developing epilepsy compared with those without early seizures [6,9,16,17]. Evidence from large pediatric cohort studies further corroborates acute seizures as a major and independent predictor of PSE [4,16,17]. A recent systematic review and meta-analysis of pediatric stroke cohorts demonstrated that acute symptomatic seizures confer an almost fourfold increased risk of subsequent PSE, highlighting the critical role of early seizure activity in pediatric epileptogenesis after ischemic injury [8]. The timing of seizure occurrence also appears to be clinically relevant. Several studies have shown that seizures occurring within the first hours after stroke onset are associated with the highest risk of subsequent epilepsy, suggesting that severe early cortical hyperexcitability may represent an early marker of epileptogenic network dysfunction [9,10,15,18].

Status epilepticus during the acute phase of stroke has also been identified as an important predictor of subsequent epilepsy. As the most severe form of acute symptomatic seizure activity, it likely reflects a combination of extensive cortical injury, greater stroke severity, and marked neuronal hyperexcitability. Several pediatric cohort studies have consistently shown that children who present with acute status epilepticus are at a substantially higher risk of developing PSE than those who experience isolated seizures, supporting its role as a marker of heightened epileptogenic vulnerability [6,8,16]. These findings support the concept of a dose–response relationship between early seizure burden and long-term epileptogenesis, whereby prolonged or recurrent seizure activity may further contribute to maladaptive network remodeling and the development of chronic epilepsy.

#### 3.3.2. Age at Stroke Onset

Younger age at stroke onset has been associated with an increased risk of PSE, particularly in infants [1,3,14,16]. This association is commonly attributed to developmental factors, including immature inhibitory networks, heightened synaptic plasticity and increased excitatory neurotransmission in the developing brain, which may lower the seizure threshold after focal ischemic injury [4,6]. In addition, strokes occurring during early life are often characterized by distinct vascular territories, etiological mechanisms, and patterns of brain injury compared with strokes occurring later in childhood, factors that may further influence long-term epileptogenic risk [19,20,21].

#### 3.3.3. Neuroimaging Characteristics

Neuroimaging features play a central role in epilepsy risk stratification. Cortical involvement has consistently emerged as one of the strongest predictors of PSE across pediatric cohorts [4,6,9,15,16]. Cortical lesions are thought to promote epileptogenesis through direct disruption of excitatory–inhibitory balance, local network hyperexcitability, and maladaptive synaptic reorganization following ischemic injury [4,6,16]. In addition to lesion location, both the extent and pattern of ischemic injury appear to influence the risk of developing epilepsy. Larger infarcts and multifocal lesions have been associated with a higher likelihood of PSE, particularly when cortical regions are affected, suggesting that widespread cortical damage may promote the development of epileptogenic networks. Importantly, several studies have reported an increased epilepsy risk even in patients with relatively small lesions when these are multifocal or involve strategically important cortical areas. These findings indicate that lesion topography may be a more relevant determinant of epileptogenesis than lesion size alone [15,16]. Anterior circulation involvement, particularly cortical infarctions within the middle cerebral artery (MCA) territory, has been associated with an increased risk of PSE, likely because these lesions disrupt highly interconnected cortical networks with greater intrinsic epileptogenic potential [4,6,9].

#### 3.3.4. EEG Characteristics

Electroencephalographic (EEG) abnormalities have been proposed as potential markers of epileptogenic risk following pediatric AIS [4,5,8]. Acute EEG abnormalities reported after pediatric AIS include focal slowing, background asymmetry or attenuation, epileptiform discharges, and electrographic seizures, particularly in patients presenting with acute symptomatic seizures or cortical involvement, reflecting the degree of cortical dysfunction induced by ischemic injury [4,5]. Early epileptiform EEG abnormalities have been proposed as potential markers of increased epileptogenic risk after pediatric AIS; however, available evidence remains heterogeneous and prospective validation is still lacking.

In addition, studies employing EEG monitoring have demonstrated that electrographic seizures and, less frequently, non-convulsive status epilepticus may occur during the acute phase of pediatric brain injury and may remain clinically unrecognized without neurophysiological monitoring [22,23]. Continuous EEG monitoring may therefore improve seizure detection and provide a more comprehensive assessment of acute seizure burden, although its specific role in predicting PSE in children remains to be fully established [22,23].

#### 3.3.5. Stroke Severity and Neurological Deficits

Greater clinical stroke severity and persistent neurological deficits are consistently associated with an increased long-term risk of PSE [2,14,15,16,17,24]. Children who subsequently develop epilepsy often exhibit poorer neurological recovery, including greater motor disability and cognitive impairment, suggesting that PSE may represent both a consequence and a marker of more severe brain injury and network disruption after childhood AIS [24].

Similarly, abnormal neurological status at hospital discharge has emerged as a robust predictor of later epilepsy, likely reflecting the combined effects of lesion burden, cortical dysfunction, and impaired recovery of neural networks during the early post-stroke period [2,16,17,24].

#### 3.3.6. Etiology and Biological Factors

The relationship between stroke etiology and PSE remains complex. Infectious, inflammatory, and arteriopathic etiologies of AIS have been associated with increased seizure risk in selected pediatric cohorts, potentially through sustained neuroinflammatory mechanisms, although findings are not fully consistent across studies [4,6].

Emerging evidence also suggests that low serum vitamin D levels may represent a potentially modifiable risk factor for PSE, with independent associations reported in pediatric populations [15,18]. Vitamin D is known to exert neuroprotective and immunomodulatory effects and may influence excitotoxicity, neuroinflammation, and epileptogenic processes following brain injury; however, the clinical relevance of these mechanisms in pediatric PSE requires further investigation.

#### 3.3.7. Genetic Susceptibility

**Figure 2:**
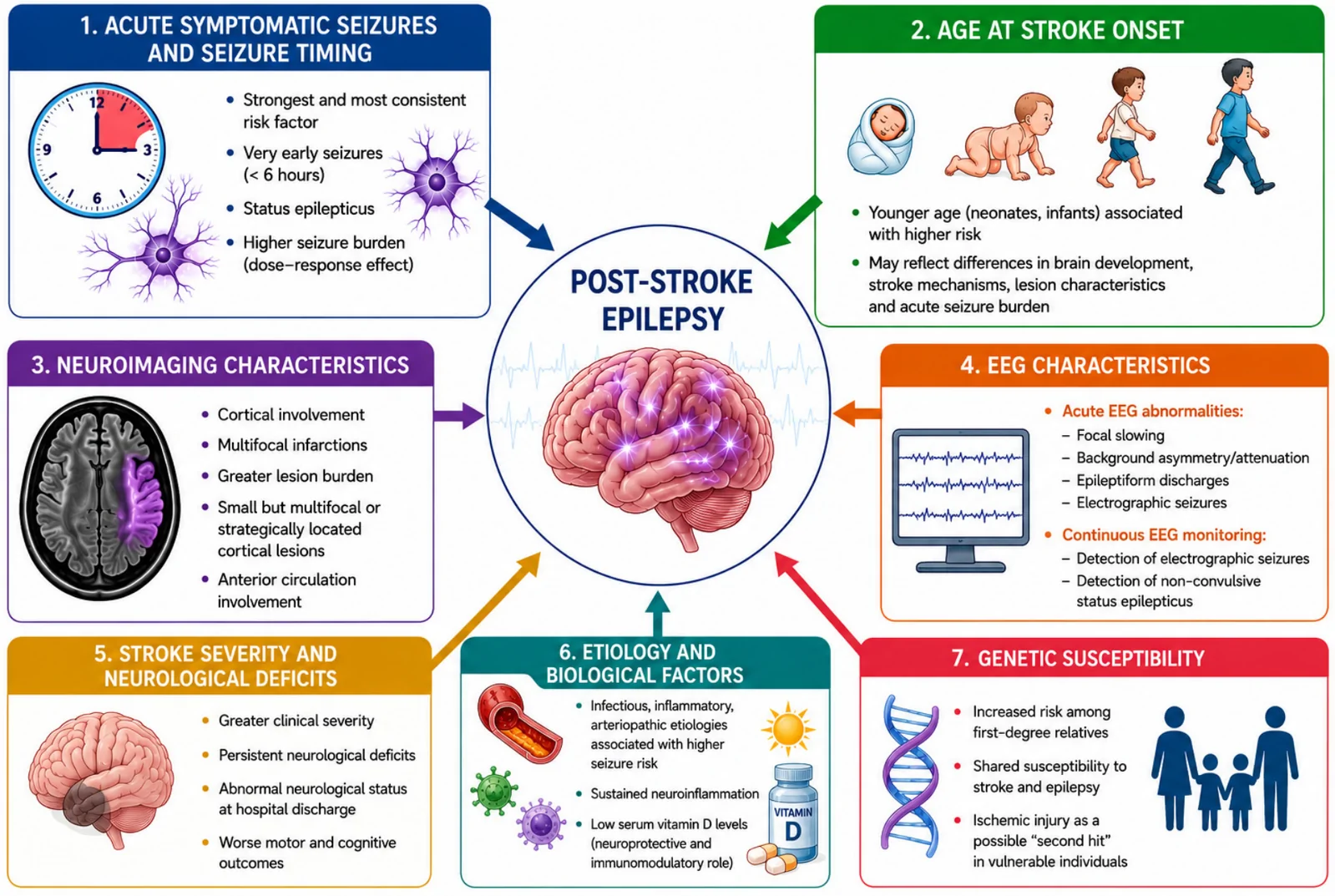
Summary of the principal risk factors for pediatric post-stroke epilepsy. The figure illustrates the main clinical, neuroimaging, EEG, biological, and genetic determinants associated with an increased risk of epilepsy following childhood stroke.

PSE has traditionally been considered a purely acquired consequence of ischemic brain injury; however, population-based evidence suggests that individual susceptibility factors may influence epileptogenesis after pediatric AIS. Sundelin et al. reported an increased risk of epilepsy among first-degree relatives of children with stroke, particularly among parents and siblings of patients who developed PSE, indicating familial aggregation beyond epilepsy alone [1]. As discussed by Beslow and colleagues, these findings challenge the concept of PSE as an exclusively acquired condition and are compatible with a model of shared susceptibility to both stroke and epilepsy, in which ischemic injury may act as a precipitating “second hit” in vulnerable individuals [25] (Figure 2).

Table 1 summarize the risk of developing PSE associated with the factors described above.

## 4. Discussion

PSE represents one of the most frequent and clinically relevant long-term sequelae of pediatric AIS.

Among the various predictors investigated to date, only a limited number of clinical factors have been consistently confirmed across independent pediatric cohorts. In contrast, several proposed biological, neuroimaging, and electrophysiological predictors remain supported by relatively small, retrospective, or methodologically heterogeneous studies and should therefore be interpreted with caution.

Original pediatric cohort studies consistently demonstrate that the risk of epilepsy remains substantially higher than in adults and persists well beyond the acute post-stroke period, although reported cumulative incidences vary widely according to study design, duration of follow up, patient characteristics, and outcome definitions [1,2,6]. Several original pediatric cohort studies have consistently shown that the temporal profile of seizures plays a critical role in determining long-term epileptogenic risk. In particular, acute symptomatic seizures, particularly those occurring within the first hours after stroke onset, represent the most consistently identified clinical predictor of PSE across pediatric cohort studies [8,9,10,15,18]. This observation suggests that severe early cortical hyperexcitability may represent not only an acute consequence of ischemic injury, but also an early marker of maladaptive epileptogenic network dysfunction.

Very early seizures are thought to reflect the complex cascade triggered by cerebral ischemia, including excitotoxic neuronal injury, disruption of ionic homeostasis, metabolic dysfunction, mitochondrial impairment, and activation of inflammatory pathways. Experimental studies suggest that excessive glutamate release, ATP depletion, oxidative stress, and impaired astrocyte–neuron metabolic coupling contribute to persistent alterations in neuronal excitability and maladaptive synaptic remodeling, thereby facilitating epileptogenesis [26]. In parallel, spreading and anoxic depolarizations increase metabolic demand in vulnerable peri-infarct tissue, potentially amplifying excitotoxic and inflammatory responses and promoting network remodeling [27]. Ischemic injury also induces a sustained neuroinflammatory response characterized by activation of microglia and astrocytes, release of pro-inflammatory cytokines and recruitment of peripheral immune cells [28]. Experimental and clinical evidence further suggests that BBB dysfunction may directly promote neuronal hyperexcitability through the extravasation of serum proteins and activation of astrocytic signaling pathways, providing an additional mechanism linking acute ischemic injury to epileptogenesis [29]. Although these mechanisms provide a biologically plausible framework linking acute ischemic injury to post-stroke epileptogenesis, direct evidence supporting their specific contribution to pediatric post-stroke epilepsy remains limited.

In the developing brain, these processes may further interact with age-dependent neuronal plasticity and the ongoing maturation of inhibitory circuits, thereby facilitating maladaptive synaptic reorganization and long-term hyperexcitability. These developmental mechanisms are particularly relevant during the neonatal period, when reduced KCC2 expression, immature chloride homeostasis, and depolarizing GABAergic signaling contribute to increased seizure susceptibility [30,31]. In contrast, in post-neonatal childhood AIS, epileptogenesis is more likely to be driven by cortical injury, excitotoxicity, neuroinflammation, and maladaptive network remodeling, although developmental factors may continue to influence individual vulnerability. Accordingly, mechanistic findings derived from neonatal stroke models should not be directly extrapolated to childhood AIS without considering age-dependent differences in brain maturation, inhibitory neurotransmission, and epileptogenic pathways. Original observational studies have consistently shown a dose–response relationship between early seizure burden and subsequent epilepsy risk, with prolonged seizures and acute status epilepticus conferring the highest risk of later recurrent unprovoked seizures [6,15,16]. These findings support the hypothesis that sustained early neuronal hyperexcitability and more severe cortical dysfunction may contribute to progressive epileptogenic network remodeling after pediatric stroke. Furthermore, prospective population-based studies suggest that the epileptogenic impact of acute seizures may evolve over time, with limited predictive value during early follow-up but significantly increased association with epilepsy at longer follow-up intervals, underscoring the progressive nature of post-stroke network remodeling in the developing brain [17].

Younger age at stroke onset has been associated with an increased risk of PSE in several cohorts, although interpretation of this finding is complicated by the frequent inclusion of neonatal and childhood stroke populations within the same studies [3,14,15].

A major limitation of the available literature is that several original cohort studies combine neonatal, presumed perinatal, and childhood stroke populations without performing age-stratified analyses [3,16]. This methodological heterogeneity complicates the interpretation of epilepsy risk because neonatal and childhood AIS differ substantially in developmental neurobiology, seizure susceptibility, underlying etiologies, and mechanisms of epileptogenesis [19,20,21]. As a result, combining these populations within the same analyses may mask age-specific risk factors and partially explain the wide variability in reported epilepsy rates across studies.

Neonates exhibit distinct developmental neurobiology characterized by enhanced excitatory neurotransmission, immature inhibitory GABAergic systems, and unique patterns of cortical connectivity, all of which influence both seizure susceptibility and long-term epileptogenic potential. In addition, neonatal strokes are frequently related to perinatal hemodynamic disturbances, placental disorders, or perinatal thrombosis, whereas childhood strokes more commonly involve arteriopathies, cardiac disease, inflammatory disorders, or prothrombotic conditions [19,20,21]. Consequently, combining these populations within single analyses may obscure age-specific predictors of epileptogenesis and contribute significantly to the wide variability in reported cumulative incidences of PSE across studies.

Despite these limitations, more recent cohort studies that separately analyzed neonatal and childhood stroke suggest that both shared and age-specific mechanisms contribute to epileptogenesis. Across pediatric age groups, acute symptomatic seizures, cortical involvement, multifocal infarctions, and abnormal neurological status at hospital discharge consistently emerge as the strongest independent predictors of PSE [16]. However, the impact of these factors may differ across developmental stages, suggesting that epileptogenesis after pediatric stroke is not a uniform process but rather one that is influenced by brain maturation and age-related biological factors. These observations underscore the importance of age-stratified analyses and caution against considering younger age as an independent risk factor without accounting for the underlying clinical and neuroimaging characteristics of the stroke.

An important limitation of the available evidence is the substantial heterogeneity across pediatric stroke studies, which limits the direct comparability of reported incidence estimates and risk factors.

Studies differ in patient age and composition, with some cohorts combining neonatal, presumed perinatal, and childhood stroke populations, lesion characteristics, duration of follow-up, and definitions and ascertainment of post-stroke epilepsy.

In addition, much of the available evidence derives from retrospective, single-center studies with relatively small sample sizes, limiting the generalizability of their findings. Consequently, although several clinical and neuroimaging factors have been consistently associated with PSE, the strength and independence of these associations should be interpreted cautiously, particularly for factors supported by a limited number of heterogeneous studies.

Neuroimaging findings play a key role in assessing epilepsy risk after pediatric AIS. Among neuroimaging features, cortical involvement is the most consistently identified predictor of post-stroke epilepsy (PSE) following childhood AIS, as demonstrated by original pediatric cohort studies [4,6,9,15,16]. Cortical injury is thought to increase epileptogenic risk by disrupting both local cortical circuits and large-scale functional brain networks, thereby promoting maladaptive synaptic remodeling and persistent neuronal hyperexcitability. Moreover, cortical lesions may directly impair the balance between excitatory and inhibitory neuronal activity while altering long-range structural and functional connectivity, ultimately facilitating the development of epileptogenic networks.

Likewise, infarctions affecting the anterior circulation, particularly within the middle cerebral artery territory, have been linked to an increased risk of epilepsy, probably owing to the involvement of highly interconnected cortical regions responsible for sensorimotor, language, and higher-order cognitive functions [4,6,9]. These lesions may facilitate widespread cortical network dysfunction and maladaptive synaptic reorganization, thereby promoting chronic hyperexcitability. Beyond lesion location, the extent and spatial distribution of ischemic injury also appear to substantially influence epileptogenic risk. Several studies have shown that multifocal infarctions and greater lesion burden are associated with higher rates of PSE, particularly when cortical regions are involved, suggesting a cumulative effect of distributed cortical injury on epileptogenic network remodeling, although direct mechanistic evidence in pediatric populations remains limited [2,6,7,24]. Indeed, emerging evidence indicates that even relatively small infarcts may confer significant epilepsy risk when lesions are multifocal or strategically located within highly interconnected cortical regions, emphasizing the importance of lesion topology and network involvement rather than infarct volume alone [15,16]. These observations support the hypothesis of a network-based model of post-stroke epileptogenesis in childhood, in which distributed cortical injury, altered interhemispheric connectivity, and maladaptive large-scale network reorganization may contribute to the progressive development of chronic cortical hyperexcitability and recurrent unprovoked seizures. However, although this model provides a biologically plausible framework, direct evidence in childhood AIS remains limited and is largely inferred from experimental studies and adult stroke populations.

Stroke etiology may also influence epileptogenic risk after pediatric AIS, although the available evidence remains heterogeneous. Original pediatric cohort studies suggest that inflammatory, infectious, and arteriopathic stroke mechanisms—particularly focal cerebral arteriopathy (FCA)—may be associated with an increased risk of PSE, potentially reflecting the contribution of persistent neuroinflammatory activation, blood–brain barrier dysfunction, and ongoing vascular injury to epileptogenesis [4,8,16]. In particular, FCA has emerged as one of the etiological subtypes most frequently associated with PSE, although this association is based primarily on retrospective studies and requires confirmation in larger prospective cohorts. Mechanistically, inflammatory arteriopathies may promote chronic cortical instability through recurrent ischemic injury, endothelial dysfunction, blood–brain barrier disruption, and sustained inflammatory signaling.

Interestingly, despite representing one of the most common etiological categories of childhood AIS, congenital and acquired cardiac disorders have not been consistently identified as independent predictors of PSE in pediatric cohorts. Any apparent association is likely mediated by stroke-related factors, including cortical involvement, lesion burden, multifocal infarctions, and recurrent ischemic events [2,4,14].

Interpretation of the current evidence is further complicated by the inclusion of mixed cerebrovascular populations in several pediatric cohorts and meta-analyses, including both arterial ischemic and hemorrhagic stroke without separate subgroup analyses. Given the important differences in neurobiological mechanisms, acute seizure propensity, and long-term epileptogenic pathways between ischemic and hemorrhagic brain injury, this heterogeneity likely represents a significant source of variability across studies.

Although EEG abnormalities have been proposed as potential biomarkers of epileptogenic risk, the available pediatric evidence remains limited and heterogeneous, precluding definitive conclusions regarding their independent prognostic value [4,5,8]. Original pediatric studies evaluating EEG after AIS remain relatively limited and heterogeneous, with important differences in acquisition protocols, timing of recording, and availability of continuous EEG monitoring. Consequently, EEG abnormalities have not been consistently identified as independent predictors of PSE across pediatric cohorts. This variability was also highlighted by the recent systematic review by Alqahtani and Makke, in which EEG findings could not be confirmed as independent predictors of epilepsy [8].

Continuous EEG monitoring may improve the detection of electrographic-only seizures and non-convulsive status epilepticus, particularly in critically ill children with persistent altered mental status or unexplained encephalopathy, in whom clinical seizure recognition may be challenging [14,22,24]. This issue is particularly relevant in pediatric stroke because unrecognized electrographic seizures and non-convulsive status epilepticus may increase overall seizure burden and potentially contribute to worse neurological outcomes [23,32]. Nevertheless, systematic cEEG monitoring has not been consistently implemented in most pediatric stroke cohorts, limiting accurate estimation of electrographic seizure burden and its relationship with long-term epileptogenesis. In children with acute AIS, cEEG should be considered in the presence of persistent impairment of consciousness, failure to return to neurological baseline after a clinical seizure, suspected non-convulsive status epilepticus, recurrent subtle paroxysmal events of uncertain nature, ongoing status epilepticus, or when sedation or pharmacological paralysis may obscure clinical seizure activity [22]. Although at least 24 h of monitoring is generally recommended in critically ill patients, the optimal duration and independent prognostic value of electrographic seizure burden specifically in childhood AIS remain uncertain because cEEG has not been systematically implemented across pediatric stroke cohorts [8,14,22]. Importantly, the prognostic value of electrographic seizure burden for the subsequent development of PSE remains uncertain because cEEG use, timing, and duration have varied considerably across pediatric stroke cohorts. Therefore, prospective studies using standardized monitoring protocols are needed to determine whether electrographic seizure burden independently predicts long-term epileptogenesis. Beyond seizure detection, EEG may also provide insight into mechanisms of network dysfunction after ischemic injury. Persistent focal slowing, abnormal background organization, and epileptiform discharges may reflect disrupted cortical connectivity, impaired maturation of neuronal networks, and maladaptive synaptic reorganization within structurally injured regions, processes that are thought to contribute to post-stroke epileptogenesis [7,12].

Greater stroke severity and persistent neurological deficits have been consistently identified as robust clinical predictors of PSE following childhood AIS [14,15,16,24]. These associations likely reflect more extensive and functionally disruptive brain injury, serving as indirect markers of widespread cortical damage, impaired network recovery, and increased epileptogenic potential.

Children who develop PSE consistently experience poorer long-term neurological outcomes than those who remain seizure-free, suggesting that epileptogenesis may represent both a consequence and a marker of more severe underlying brain injury and network disruption after pediatric AIS [2,3,17,24].

Although PSE has been consistently associated with poorer overall functional outcomes, its independent contribution to long-term cognitive, neuropsychological, behavioral, and academic impairment remains insufficiently characterized. Most pediatric stroke studies have focused primarily on global neurological disability and functional status, whereas detailed longitudinal assessments of cognition, executive functions, psychosocial well-being, and educational achievement remain scarce [33]. Consequently, the extent to which PSE independently contributes to long-term neuropsychological sequelae, beyond the effects of the initial ischemic injury, remains an important area for future investigation.

In addition to established clinical and neuroimaging risk factors, emerging population-based data suggest that inherited biological susceptibility may contribute to the risk of PSE; however, direct evidence from pediatric genetic studies remains limited, and these findings require further validation. Large nationwide registry studies have reported an increased prevalence of epilepsy among first-degree relatives of affected children, supporting the hypothesis that inherited biological factors may modify individual susceptibility to epileptogenesis after stroke. These findings raise the prospect that genetic factors, or other inherited mechanisms influencing neuronal excitability and brain resilience, may contribute to the risk of developing epilepsy after pediatric stroke [1]. In this context, ischemic injury may act as a precipitating factor capable of unmasking latent epileptogenic predisposition in susceptible individuals [25]. These observations are consistent with emerging concepts of precision medicine, in which genetic susceptibility may interact with acquired brain injury to determine individual epileptogenic risk.

Emerging evidence suggests that modifiable biological factors may also contribute to epileptogenic risk after pediatric stroke. Low serum vitamin D levels have recently been proposed as a potential risk factor, but current evidence is based on a limited number of observational studies and should be considered preliminary pending confirmation in prospective multicenter cohorts [15,18]. Although the underlying mechanisms remain incompletely understood, vitamin D is known to exert neuroprotective, anti-inflammatory, antioxidant, and immunomodulatory effects, and may influence neuronal excitability and post-ischemic network remodeling. These observations raise the possibility that part of the biological vulnerability to post-stroke epileptogenesis could be potentially modifiable.

Despite these advances, important knowledge gaps remain regarding the complex interplay between genetic susceptibility, inflammatory pathways, metabolic factors, and ischemia-induced network reorganization in pediatric PSE. Future prospective multicenter studies will be essential to better define the mechanisms underlying epileptogenesis and improve risk stratification.

Overall, the current evidence supports acute symptomatic seizures, status epilepticus, cortical involvement, and persistent neurological deficits as the most robust clinical predictors of PSE. By contrast, the prognostic value of lesion burden, EEG abnormalities, inflammatory biomarkers, vitamin D deficiency, genetic susceptibility, and network-based imaging markers remains promising but requires confirmation in larger prospective pediatric studies.

Finally, as this manuscript is a narrative review, the selection and synthesis of the available literature are inherently subject to methodological limitations, and no quantitative pooling or formal comparison of effect estimates across studies was performed. Prospective multicenter studies using standardized definitions, follow-up protocols, and outcome ascertainment are needed to better establish the incidence of PSE and validate potential risk factors across pediatric populations.

### 4.1. Clinical Implications

The consistently high incidence and long-term persistence of epilepsy risk after pediatric AIS underscore the need for prolonged neurological follow-up [1,2,6,8]. Data from pediatric cohorts indicate that acute symptomatic seizures may predict PSE over longer time horizons, even when short-term prediction is less robust, supporting the need for sustained follow-up beyond the early months after stroke [6,8,17].

Children presenting with early seizures, cortical involvement, severe stroke, multifocal infarctions, focal cerebral arteriopathy, or persistent neurological deficits should be considered at particularly high risk and may benefit from closer surveillance, including targeted EEG monitoring [2,6,8,15,16,17,24]. Improved risk stratification may facilitate family counseling, individualized follow-up strategies, and the design of future interventional studies aimed at preventing epileptogenesis.

Importantly, pediatric cohorts demonstrate that PSE is associated with worse neurological, motor, and cognitive outcomes after childhood AIS, further emphasizing the clinical value of early identification and longitudinal care pathways [2,24]. The identification of potentially modifiable risk factors may offer opportunities to improve long-term outcomes after pediatric stroke. In particular, strategies such as optimizing the management of acute symptomatic seizures and addressing vitamin D deficiency have emerged as potential targets for intervention, although prospective evidence supporting their effectiveness remains limited [8,15,18].

Finally, growing evidence suggests that multimodal approaches integrating clinical features, EEG findings, neuroimaging markers, and biological factors may improve individualized risk prediction and support the development of future anti-epileptogenic strategies in pediatric stroke survivors [8,12].

Future studies integrating clinical, neuroimaging, and genetic data may help clarify individual vulnerability to PSE and improve long-term risk stratification. In particular, multimodal approaches that integrate advanced neuroimaging, electrophysiological biomarkers, inflammatory markers, and predictive computational models may improve the early identification of children at greatest risk of developing PSE. Advanced neuroimaging techniques, including structural and functional network-based MRI analyses, may provide valuable insights into patterns of cortical disconnection and maladaptive network reorganization that contribute to epileptogenesis following pediatric AIS. Similarly, quantitative EEG features, electrographic seizures, and early epileptiform abnormalities may represent promising biomarkers of cortical hyperexcitability and long-term epilepsy risk, although prospective pediatric studies using standardized EEG protocols remain limited. Growing evidence also supports the potential contribution of inflammatory and metabolic pathways to post-stroke epileptogenesis. Future investigations should therefore explore the role of circulating inflammatory biomarkers, blood–brain barrier dysfunction, genetic susceptibility, and modifiable biological factors such as vitamin D deficiency in determining individual epileptogenic vulnerability. Integration of genomic and molecular profiling may further improve understanding of shared susceptibility mechanisms linking stroke and epilepsy in childhood.

Prospective multicenter pediatric stroke registries incorporating standardized EEG acquisition, advanced neuroimaging protocols, and biological sampling will be essential to validate candidate biomarkers of epileptogenesis and to improve the reproducibility and generalizability of risk prediction models across pediatric populations.

In parallel, emerging artificial intelligence and machine learning approaches may facilitate development of predictive models integrating clinical variables, lesion topology, EEG characteristics, and multimodal imaging data to improve early risk stratification and individualized follow-up strategies. Ultimately, better characterization of epileptogenic pathways after stroke in children may support the development of targeted anti-epileptogenic interventions aimed at preventing or reducing long-term epilepsy burden in high-risk children.

### 4.2. Limitations

The findings summarized in this review should be interpreted in light of several limitations. First, most of the available evidence originates from retrospective observational studies with relatively small sample sizes, limiting statistical power and reducing the generalizability of the reported findings. Second, substantial heterogeneity exists across studies regarding patient age, stroke etiology, neuroimaging characteristics, follow-up duration, and definitions of acute symptomatic seizures and post-stroke epilepsy, making direct comparisons challenging. Third, EEG acquisition protocols, including the use and duration of continuous EEG monitoring, were inconsistently applied, likely resulting in under-recognition of electrographic seizures and limiting evaluation of their prognostic significance. Furthermore, most proposed biological, electrophysiological, and neuroimaging biomarkers remain supported by limited observational evidence and require prospective validation. Finally, as this is a narrative review, a formal systematic methodology, risk-of-bias assessment, and quantitative meta-analysis were not performed; therefore, some degree of selection bias cannot be excluded.

## 5. Conclusions

PSE is one of the most common and clinically significant long-term complications of pediatric AIS. Compared with adults, children face a substantially higher and more persistent risk of developing epilepsy, with long-term follow-up studies indicating that up to one-quarter of pediatric stroke survivors may eventually experience unprovoked seizures. Among the identified risk factors, acute symptomatic seizures, particularly those occurring very early after stroke and episodes of status epilepticus, consistently emerge as the strongest predictors of subsequent epilepsy. Additional contributors include younger age at stroke onset, cortical involvement, multifocal or extensive infarctions, greater stroke severity, and persistent neurological impairment.

These findings highlight the importance of early recognition of children at elevated risk and support the need for structured long-term neurological surveillance as an integral component of pediatric stroke care. Future research should focus on improving individualized risk prediction through multimodal approaches that integrate clinical characteristics, advanced neuroimaging, electrophysiological biomarkers, molecular signatures, and genetic susceptibility factors. A deeper understanding of the mechanisms driving epileptogenesis in the developing brain may ultimately pave the way for precision medicine strategies and the development of targeted interventions aimed at preventing epilepsy after childhood AIS.

Ultimately, the ability to identify children at highest risk before the onset of epilepsy may represent a crucial step toward precision prevention and the future implementation of effective anti-epileptogenic therapies following pediatric AIS.

## Figures and Tables

**Table 1:** Association of risk factors with the development of PSE.

Risk Factor	Association with PSE	Key References
Acute symptomatic seizures	Strongly increased risk	[4,5,6,8,9,10,16,17,18]
Very early seizures (<6 h)	Strongly increased risk	[8,9,10,15,18]
Acute Status epilepticus	Strongly increased risk	[6,8,16,18]
Younger age at stroke onset	Increased risk	[1,3,8,14,16]
Cortical involvement	Strongly increased risk	[4,6,8,9,15,16]
Middle Cerebral Artery (MCA) involvement	Increased epileptogenic potential	[4,6,8,9]
Focal cerebral arteriopathy (FCA)	Increased risk	[8,16]
Multifocal infarctions	Increased risk	[15,16]
Large infarct burden	Increased risk	[15,16]
Greater stroke severity	Increased risk	[6,14,15,16]
Persistent neurological deficits	Increased long-term risk	[6,14,15,16,24]
Low serum vitamin D levels	Increased risk (emerging factor)	[15,18]
Genetic susceptibility	Possible increased risk	[25]

## Data Availability

No new data were created or analyzed in this study. Data sharing is not applicable to this article.

## Abbreviations

The following abbreviations are used in this manuscript:

PSE	Post-Stroke Epilepsy
AIS	Arterial Ischemic Stroke
FCA	Focal Cerebral Arteriopathy
ILAE	International League Against Epilepsy
EEG	Electroencephagraphic
cEEG	continuous Electroencephalographic
